# Rhabdomyolysis Induced by Irbesartan‐Hydrochlorothiazide in an Elderly Patient

**DOI:** 10.1002/ccr3.71295

**Published:** 2025-10-15

**Authors:** Wei Yang, Ju Luo, Wei Chen, Biao Peng

**Affiliations:** ^1^ Department of Gerontology Yueyang County People's Hospital Chengguan China; ^2^ Department of Gerontology Changsha Central Hospital Changsha China

**Keywords:** gerontology, hyponatremia, irbesartan‐hydrochlorothiazide, rhabdomyolysis

## Abstract

A 90‐year‐old male with hypertension and diabetes presented with acute limb weakness and dizziness. Laboratory tests revealed severe hyponatremia (Na^+^ 111.6 mmol/L) and rhabdomyolysis (CK 1771 U/L), which occurred after the recent initiation of irbesartan‐hydrochlorothiazide for edema. The patient's symptoms resolved, and the laboratory test results normalized after the drug was discontinued and the patient's sodium concentration was corrected with hypertonic saline. This case highlights hydrochlorothiazide‐induced hyponatremia as a rare trigger for rhabdomyolysis in elderly patients with comorbidities.

**Summary**

- Herein, we report an underrecognized case of hydrochlorothiazide‐induced severe hyponatremia causing rhabdomyolysis.
- In elderly patients being treated with thiazides, monitoring for hyponatremia is necessary as hyponatremia can precipitate rhabdomyolysis in addition to electrolyte imbalances such as hypokalemia.
- Early recognition prevents complications.

## 1 Patient Information

A 90‐year‐old male with a 3‐day history of “limb weakness” presented at our hospital on November 4, 2024. Electrolyte tests revealed a sodium concentration of 113.3 μmol/L, leading to a diagnosis of hyponatremia.

## 2 History of Present Illness

The patient experienced unexplained limb weakness 3 days prior, primarily in the proximal muscles of both the upper and lower limbs, without significant muscle pain. The main symptoms were difficulty walking and raising his arms. The frequency of urination was generally normal, as was the urine volume, which was approximately 1000 mL per day. However, the color of the urine was dark brown. Additional symptoms included mild dizziness, poor appetite, and intermittent cough without sputum. There was no fever, chest tightness, nausea, or vomiting. His general condition was stable.

## 3 Past Medical History

The patient had a history of Grade 3 hypertension (very high‐risk group) and Type 2 diabetes. He had been on long‐term treatment with nifedipine (antihypertensive) and gliclazide (antidiabetic), with satisfactory control of blood pressure and glucose levels. One week prior, owing to bilateral leg edema, nifedipine was replaced with irbesartan‐hydrochlorothiazide.

## 4 Physical Examination

The patient's vital signs were as follows: temperature 36.4°C, pulse 70 bpm, respiration 20/min, BP 142/75 mmHg. The patient was alert and cooperative. Examinations of his skin and neck were unremarkable. Lung auscultation revealed clear breath sounds without rales or friction rubs. His heart sounds were normal without murmurs or friction rubs. The abdominal examination revealed no abnormalities. No edema was observed. His muscle strength was Grade 3/4 for the upper and lower limbs. Mild tenderness in the proximal lower limbs was detected, and the patient's muscle tone was normal. Coordination was normal. Skin color, temperature, and peripheral pulses were normal. All the peripheral pulses of the affected limbs were palpable.

## 5 Laboratory and Imaging Findings

### 5.1 Initial Results (Nov 4)

Electrolyte Levels: Sodium 111.6 mmol/L, Potassium 4.06 mmol/L, Chloride 80.3 mmol/L. Renal Function Levels: Urea nitrogen 7.6 mmol/L, Creatinine 118 μmol/L, Uric acid 225 μmol/L. Cardiac Enzyme Levels: Myoglobin 822 ng/mL, CK 818 U/L, CK‐MB 65.5 U/L, LDH 213 U/L. Complete Blood Count: WBC Count 12.09 × 10^9^/L, RBC Count 3.88 × 10^12^/L, Hemoglobin Concentration 119 g/L, Platelet Count 299 × 10^9^/L. Imaging: Head and chest CT showed multiple chronic lacunar infarctions, brain atrophy, mild pulmonary fibrosis, and partial lung infection.

### 5.2 Follow‐Up Testing

**FIGURE 1:**
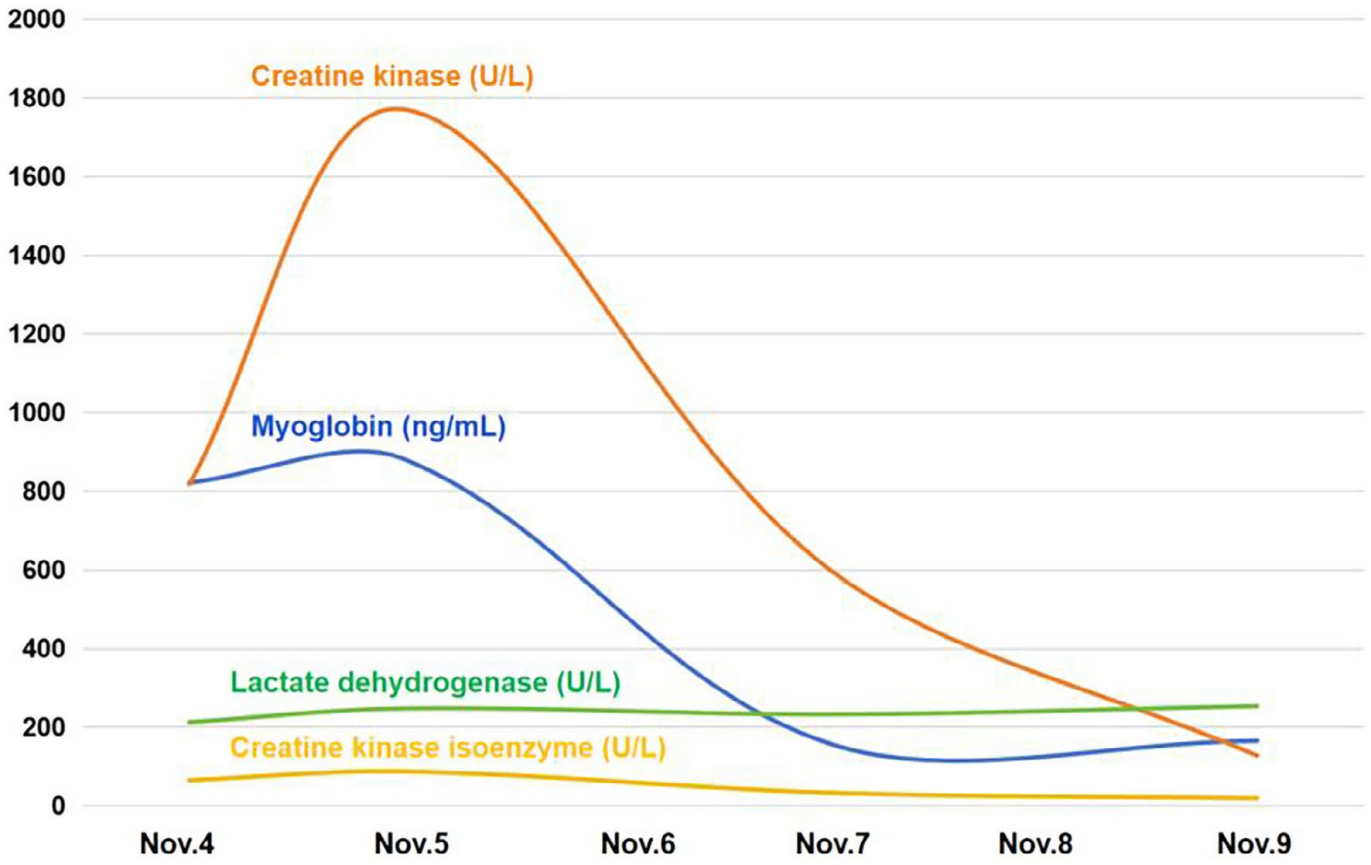
Trends in the myocardial enzyme spectra.

**FIGURE 2:**
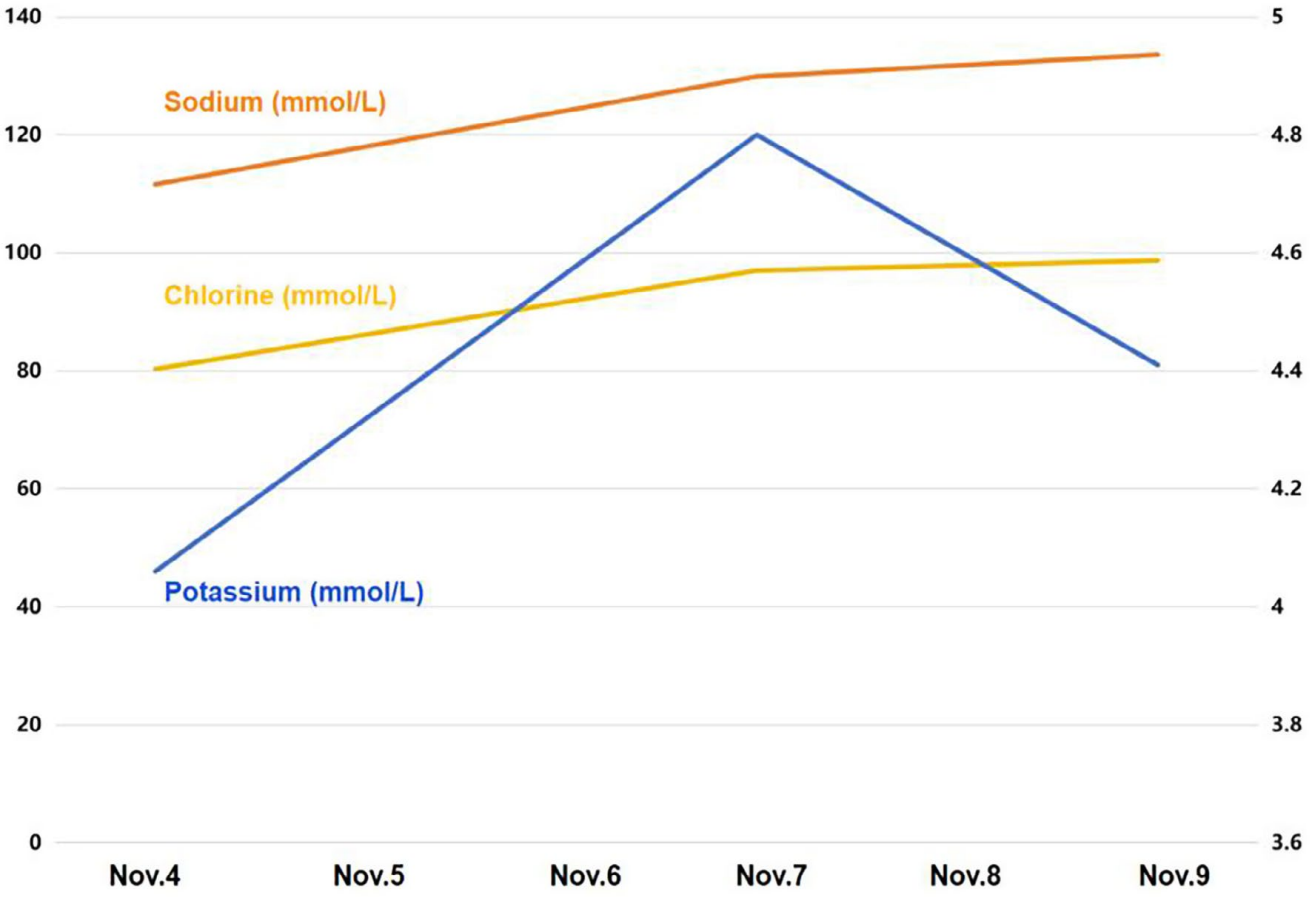
Trends in electrolyte changes.

On November 5: CK 1771 U/L, Myoglobin 883 ng/mL, Sodium 129.9 mmol/L. On November 9: CK 128 U/L, Myoglobin 166 ng/mL, Sodium 133.6 mmol/L. The results of the patient's examination are shown in Table 1, the trends in the myocardial enzyme spectra are shown in Figure 1, and the trends in the changes in electrolytes are shown in Figure 2.

**TABLE 1:** The patient's examination results.

	November 4	November 5	November 7	November 9
White blood cell (10^9^/L)	12.09	10.03	8.10	
Red blood cell (10^12^/L)	3.88	3.37	3.66	
Hemoglobin (g/L)	119.00	106.00	107	
Platelet (10^9^/L)	299.00	294.00	371.00	
Myoglobin (ng/mL)	822.00	883.00	158.40	166.00
Creatine kinase (U/L)	818.00	1771.00	600.00	128.00
Creatine kinase isoenzyme (U/L)	65.50	88.00	33.70	19.70
Lactate dehydrogenase (U/L)	213.00	248.00	233.00	254.00
Potassium (mmol/L)	4.06		4.80	4.41
Sodium (mmol/L)	111.60		129.90	133.60
Chlorine (mmol/L)	80.30		97.0	98.70
Urea nitrogen (mmol/L)	7.60			
Creatinine (μmol/L)	118.00			
Uric acid (μmol/L)	225.00			

## 6 Hospital Course

The patient reported weakness in the limbs and poor appetite during the emergency visit. An emergency physician was concerned about cerebrovascular accidents and pulmonary infections; thus, a CT scan was performed, and antibiotics were prescribed. In the emergency department, the patient was treated with cefuroxime for infection, gliclazide and dapagliflozin for glucose control, and hypertonic saline to correct hyponatremia.

After the patient was transferred to the geriatric department, we discontinued irbesartan‐hydrochlorothiazide and switched to nifedipine for blood pressure control. We continued administering hypertonic saline to correct the hyponatremia. Given the patient's poor appetite and that regular monitoring of his blood sugar levels revealed that they were normal, we temporarily discontinued the medication for hypoglycemia. Given that the patient had no other symptoms of infection (e.g., fever or productive cough) and we compared the CT images taken 2 months ago with the current CT images, pneumonia has been ruled out; we stopped the antibiotics. Five days after admission, his muscle weakness improved significantly, and the laboratory parameters normalized.

## 7 Discussion

Rhabdomyolysis is a severe syndrome caused by skeletal muscle injury, leading to the release of intracellular contents into the bloodstream. Common complications include acute kidney injury. It is diagnosed on the basis of elevated serum creatine kinase (CK) levels, which are typically 5 times the normal upper limit (> 1000 U/L) [1]. In general, the common causes of rhabdomyolysis are trauma (crush syndrome), fatigue (overexercise, epilepsy), muscle hypoxia (occlusive arteries), genetic defects, infections, temperature changes (high fever, low body temperature), metabolic and electrolyte disorders (low potassium), drugs and poisons (statins, heroin), and idiopathic causes [2, 3].

**FIGURE 3:**
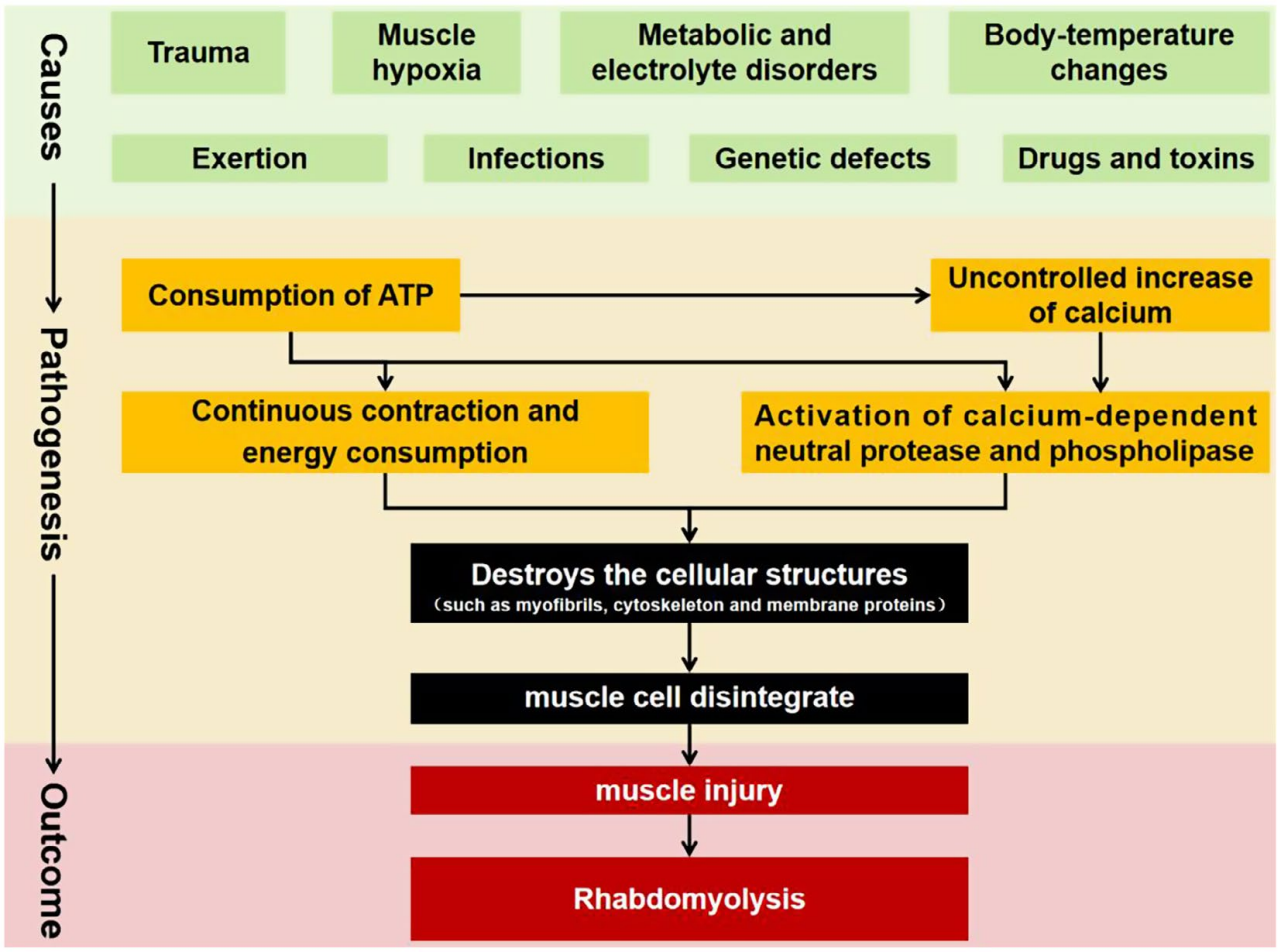
Causes and pathogenesis of rhabdomyolysis.

In addition to the direct muscle injury caused by trauma, the pathogenesis of rhabdomyolysis is mainly due to the consumption of ATP in muscle cells, which leads to an uncontrolled increase in calcium in cells. This subsequently leads to the continuous contraction and energy consumption of muscle cells, as well as the activation of calcium‐dependent neutral protease and phospholipase, ultimately destroying cellular structures such as myofibrils, the cytoskeleton, and membrane proteins. This results in the disintegration of muscle cells [4, 5]. The causes and pathogenesis of rhabdomyolysis are summarized in Figure 3.

In general, the most common drugs that cause rhabdomyolysis are statins and not irbesartan‐hydrochlorothiazide. The most common electrolyte disorder leading to rhabdomyolysis is hypokalemia rather than hyponatremia. However, in this case, the patient did not take any statins, and the serum potassium level was within the normal range. Some diseases, such as infection and diabetes, can also easily lead to rhabdomyolysis in elderly patients. In this case, the 90‐year‐old man had a history of diabetes, and his lung CT scan indicated mild infection. However, his blood sugar level was normal, and he had no other symptoms of infection. After discontinuing irbesartan‐hydrochlorothiazide and receiving hypertonic saline to correct the hyponatremia, his muscle weakness improved significantly.

In clinical practice, irbesartan‐hydrochlorothiazide is more prone to cause potassium disorders. The FLASH study included 780 patients with Grade 2 and 3 hypertension. With irbesartan‐hydrochlorothiazide monotherapy, nine cases of hypokalemia occurred, accounting for 1.2%, three cases of hyperkalemia occurred, accounting for 0.4%, and no hyponatremia was observed [6]. However, the low blood sodium concentration caused by irbesartan‐hydrochlorothiazide should also be considered. In one case report, the patient took irbesartan‐hydrochlorothiazide for an extended period and sought treatment for strokelike symptoms. The blood sodium concentration was 99 mmol/L, and the symptoms were relieved after sodium supplementation [7]. A recent study in Spain revealed that hydrochlorothiazide was the most common drug that induced hyponatremia, accounting for 28.1% (185/659), and that irbesartan was also reported to induce hyponatremia, although to a lesser extent, accounting for 1.5% (10/659) [8]. A Korean study revealed that among hypertensive patients, the 6‐year overall survival rate was better in the thiazide group than in the nonthiazide group, but the risk of hospitalization due to hyponatremia significantly increased, and the patient's outcome significantly worsened once hyponatremia occurred [9].

Electrolyte disturbances, including hypokalemia, hypophosphorus, and hypocalcemia, are common disorders that cause rhabdomyolysis [2]. Rhabdomyolysis caused by hyponatremia has been occasionally reported. For example, N'joumi Younes et al. reported a case of rhabdomyolysis caused by hyponatremia induced by olanzapine [10]. Shintaro Watanabe et al. reported a case of rhabdomyolysis induced by hyponatremia in a psychiatric patient [11]. Takeshi Komatsu et al. reported a case of isolated adrenocorticotrophin deficiency, manifested as severe hyponatremia and rhabdomyolysis [12]. In a cross‐sectional study involving 870 patients with rhabdomyolysis, severe hyponatremia (sodium concentration < 125 mmol/L) was detected in 2.1% of the patients [13]. The main mechanisms of hyponatremia leading to rhabdomyolysis are as follows: A decrease in the osmotic pressure of extracellular fluid leads to cell membrane swelling, which in turn leads to the leakage of intracellular potassium ions and causes cell membrane depolarization. This results in the release of various intracellular enzymes, such as creatine kinase, and a decrease in extracellular sodium ions, which inhibits the sodium–calcium exchange system on the cell membrane and leads to the overload of intracellular calcium ions, thus damaging muscle cells [11].

Although hyponatremia‐induced rhabdomyolysis has been reported occasionally, hyponatremia‐induced rhabdomyolysis induced by irbesartan‐hydrochlorothiazide has rarely been reported. In clinical practice, elderly patients often have other complications, such as diabetes and infection. In addition to hyponatremia caused by hydrochlorothiazide, pulmonary infection can cause inappropriate antidiuretic syndrome and lead to hyponatremia [14]. Both diabetes and some drugs used to treat diabetes, such as sulfonylureas, may induce hyponatremia [15, 16]. Thus, clinicians managing elderly patients being treated with thiazides—especially those with comorbidities—should monitor for hyponatremia, recognizing its potential to trigger rhabdomyolysis in addition to typical electrolyte imbalances such as hypokalemia.

## Author Contributions

**Wei Yang:** methodology, writing – original draft. **Ju Luo:** data curation, funding acquisition. **Wei Chen:** funding acquisition, writing – original draft. **Biao Peng:** writing – review and editing.

## Ethics Statement

This study was conducted in accordance with the Declaration of Helsinki. Written informed consent was obtained from the patient.

## Conflicts of Interest

The authors declare no conflicts of interest.

## Data Availability

The data that support the findings of this study are available from the corresponding author upon reasonable request.
